# Supplementary material for: Cell atlas of the regenerating human liver after portal vein embolization
Source: Nat Commun. 2024 Jul 11;15:5827. doi: 10.1038/s41467-024-49236-7 (PMC11239663; doi:10.1038/s41467-024-49236-7)
Supplement: Supplementary file 1 — Supplementary Information [file 41467_2024_49236_MOESM1_ESM.pdf]

Supplementary Information

Supplementary Figures

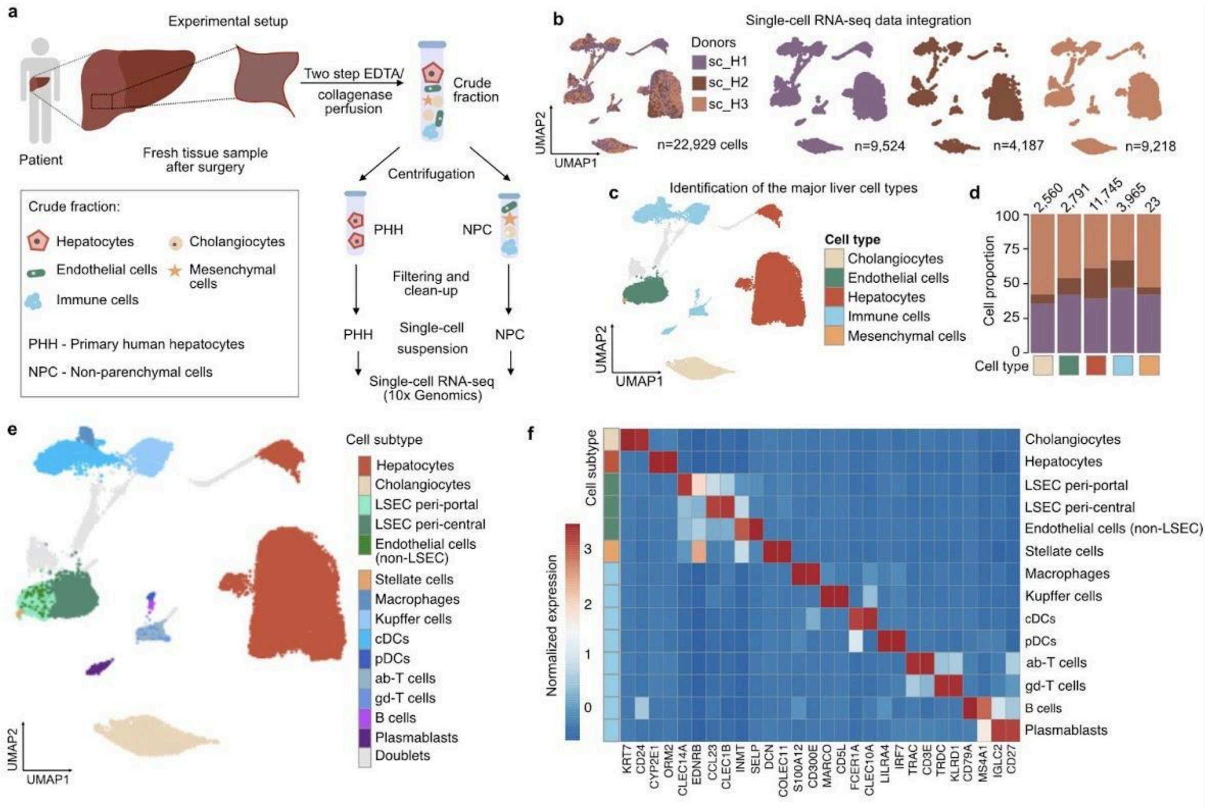

Supplementary Fig. 1 | Single-cell RNA-seq from fresh human liver control specimens reveals different cellular subtypes. **a**, Experimental schematic illustrating isolation of the primary human hepatocytes (PHH) and non-parenchymal cells (NPC) from patient-derived tissue samples (n=3). Two cellular fractions were processed and used separately to perform scRNA-seq. **b, c**, UMAP plots of scRNA-seq data from three fresh healthy (H) liver donors, colored by individual donor contributions (**b**), and by the identified major cell types (**c**). **d**, Barplots show proportions of cells from three donors across cell types, along with the absolute number of cells detected per cell type (top). **e**, UMAP showing different cell subtypes within the liver. **f**, Heatmap showing expression of cell marker genes per annotated cell subtype (genes are represented in columns, cell subtypes in rows). Source data are provided as a Source Data file for panels b,d-f.

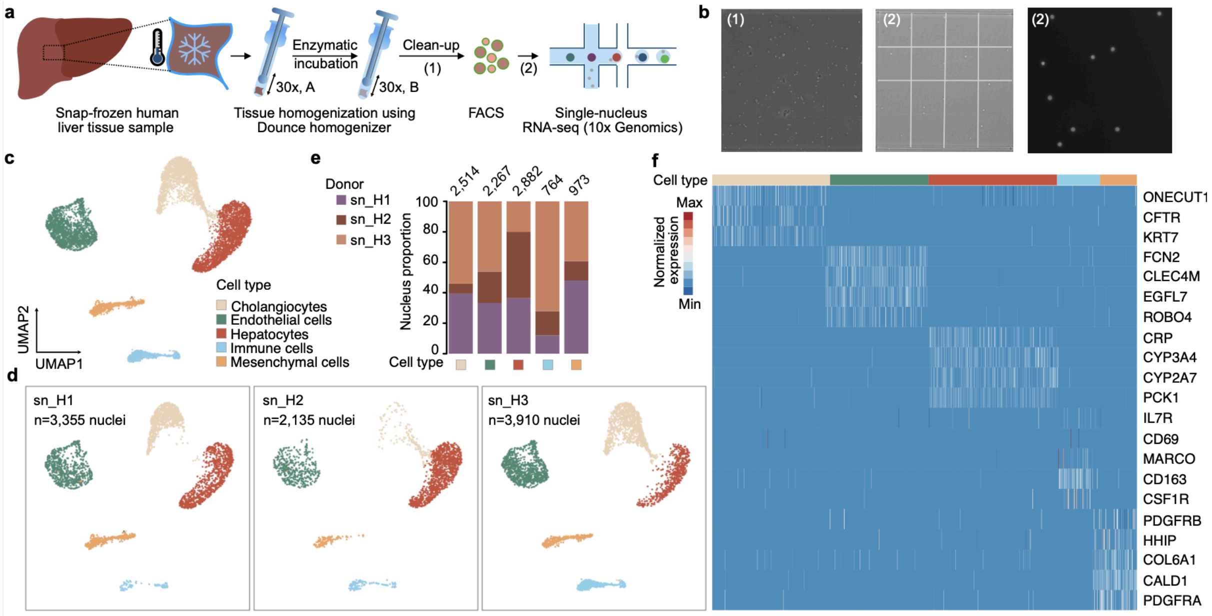

Supplementary Fig. 2 | Single-nucleus RNA-seq from snap-frozen healthy human liver specimens. **a,** Workflow illustrating experimental steps of single nucleus isolation from snap-frozen human liver tissue. **b,** Phase-contrast micrographs show representative single-nucleus suspension stained with DAPI after filtration and clean-up before (1) and (2) after FACS (10x magnification). **c, d**, UMAP plots of single-nucleus transcriptomes of three frozen healthy liver samples colored by cell type and showing merged (**c**) or individual (**d**) contributions of each donor. **e**, Barplots showing proportions of cells from three donors across identified cell types are shown together with the absolute number of cells detected per cell type (on top). **f**, Heatmap shows normalized expression of cell marker genes per annotated cell type (nuclei are represented in columns, genes in rows). Source data are provided as a Source Data file for panel c.

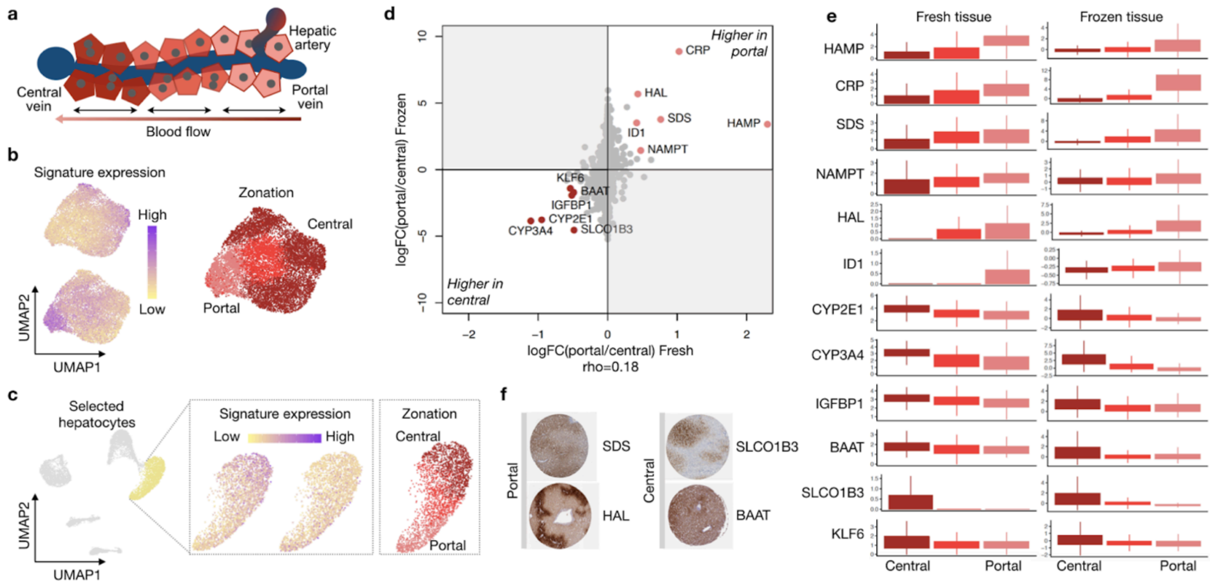

Supplementary Fig. 3 | Identification of the spatial pattern of zonation in healthy tissue hepatocytes. **a**, Schematic of portal-central axis of zonation in hepatocytes within liver lobule. **b**, UMAP plot of fresh tissue hepatocytes showing average gene expression of portal (bottom left) and central marker genes (upper left) and annotated subclusters (right) based on zonation signature expression (see Methods). **c**, UMAP plot of frozen tissue with hepatocytes showing average gene expression of portal (bottom left) and central marker genes (upper left) and annotated subclusters (right) based on the zonation signature expression (see Methods). **d**, Fold change showing differential expression in hepatocytes from portal and central subclusters in fresh (x-axis) and frozen (y-axis) tissue data (Spearman's rho=0.19, p=2.7x10^{-21}). Genes sharing the zonation pattern, i.e. genes with abs(logFC)>0.4 for fresh and abs(logFC)>1 for frozen are shown in light red (portal) and dark red (central), respectively.
e, Expression of identified genes across hepatocytes from portal and central subclusters in fresh and frozen tissue datasets. Boxplot shows the 25th, and 75th percentile (lower and upper boundary), and the whiskers indicate the 1.5x inter-quartile range. **f**, Immunostaining (Human Protein Atlas) of SDS, HAL and SLCO1B3, BAAT proteins representing portal and central zonation pattern, respectively.

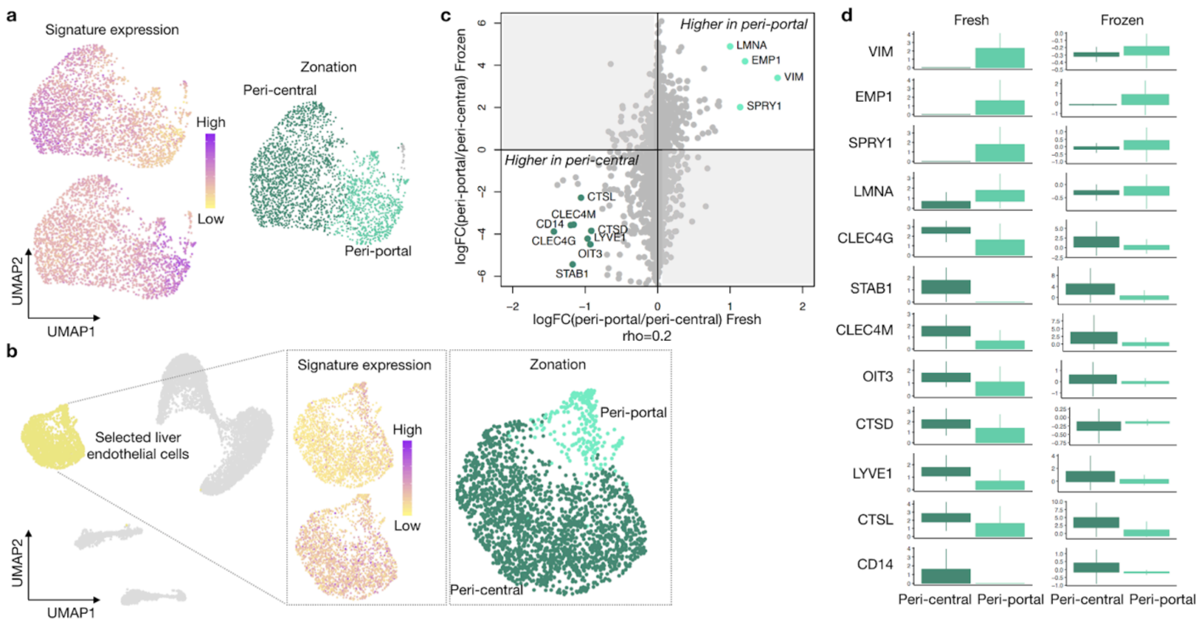

Supplementary Fig. 4 | Identification of the spatial pattern of zonation in liver sinusoidal endothelial cells (LSECs). **a**, UMAP plot of fresh tissue LSECs showing average gene expression of the previously described zonation representing genes for pericentral (upper left) and periportal (lower left) zones and accordingly assigned periportal and pericentral subclusters (right). **b**, UMAP plot of the frozen tissue showing expression signatures of periportal (upper left) and pericentral (lower left) marker genes in LSECs and according to that assigned periportal and pericentral subclusters (right). **c**, Fold change showing differential expression in LSECs from periportal and pericentral subclusters in fresh (x-axis) and frozen (y-axis) tissue data (Spearman's rho=0.2, p = 4.6×10^{-27}). Genes sharing the zonation pattern, i.e. genes with abs(logFC)>0.9 for fresh and abs(logFC)>2 for frozen are shown in light green (periportal) and dark green (pericentral), respectively. **d**, Expression of identified genes shown in panel c) across LSEC from periportal and pericentral subclusters in fresh and frozen tissue datasets. Boxplot shows the 25th, and 75th percentile (lower and upper boundary), and the whiskers indicate the 1.5x inter-quartile range.

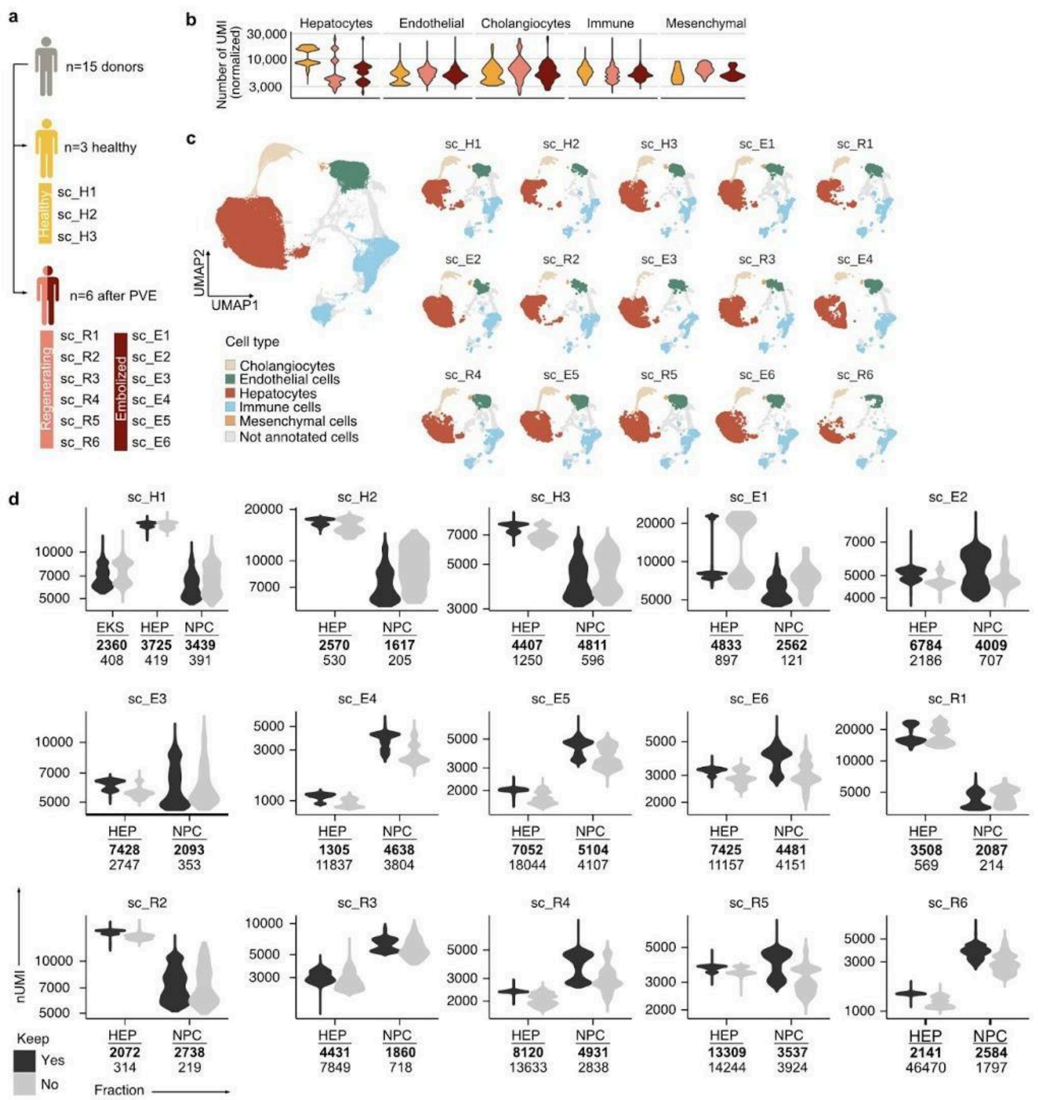

Supplementary Fig. 5 | Gene expression coverage per donor and condition. **a,** Schematic shows information on acquired liver tissues from all donors (top) including healthy (middle, yellow) and after PVE (bottom). From each donor after PVE we obtained regenerating (salmon) and embolized (dark red) conditions. sc_H, single-cell RNA-seq Healthy; sc_R, single-cell RNA-seq Regenerating; sc_E, single-cell RNA-seq Embolized**. b,** Number of detected unique molecular identifiers (UMIs) per annotated cell type**. c**, UMAP plots of scRNA-seq data for patient-derived liver tissues (n=131,961 cells) from all medical conditions and colored by major cell types are shown for all combined medical conditions (left) and for each donor individually (right). **d,** Number of kept (dark gray) and filtered out (light gray) UMIs per analyzed cellular fraction (EKS, mix of Endothelial, Kupffer and HSCs; Hep, Hepatocytes; NPC, non-parenchymal cells (see Methods)) across all samples**.** Source data are provided as a Source Data file for panels b-d.

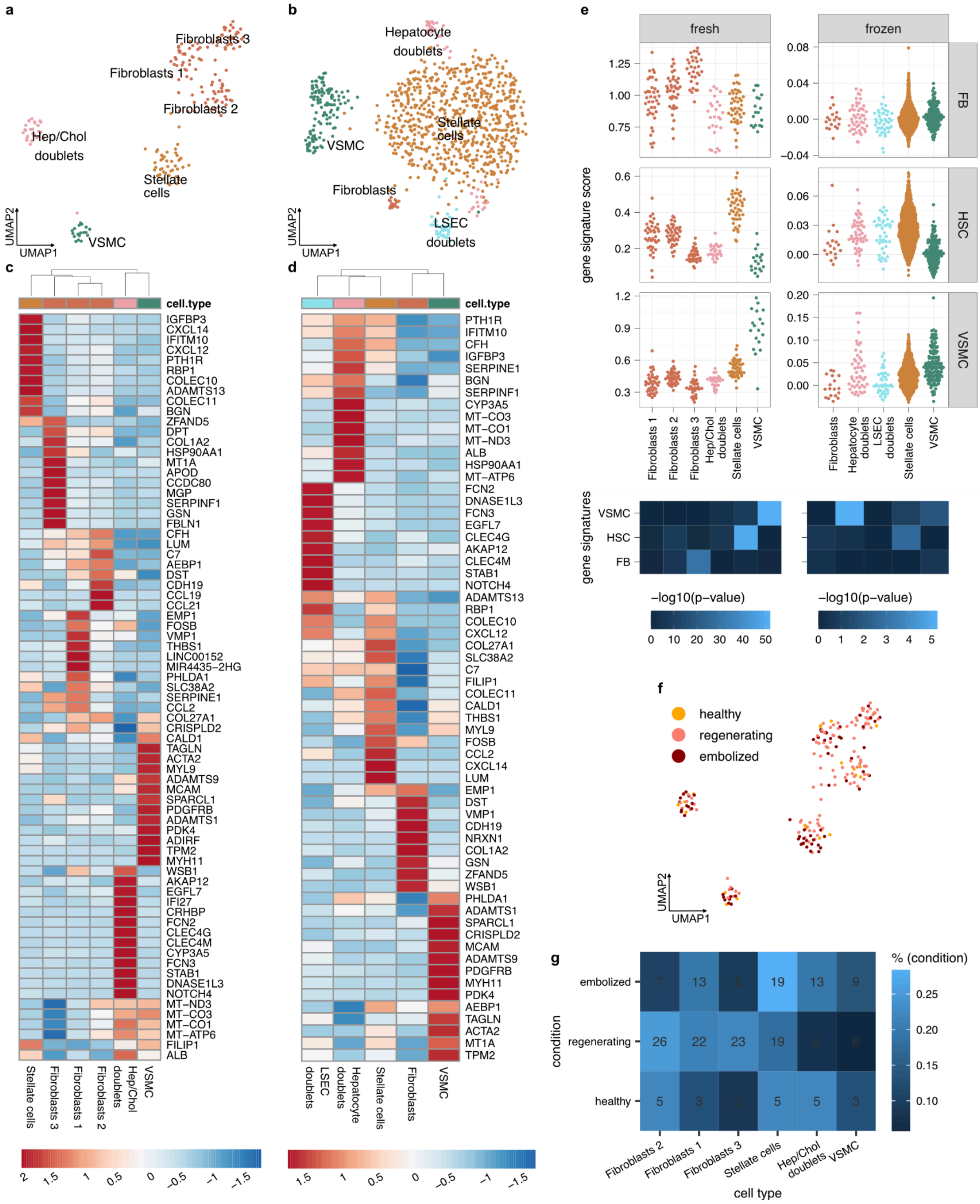

Supplementary Fig. 6 | Mesenchymal cell heterogeneity. a, UMAP plot of mesenchymal cells from healthy and post-PVE fresh samples. **b**, UMAP plot of mesenchymal cells from healthy frozen samples. **c and d**, Scaled average expression of top marker genes identified for fresh (c) and frozen (d) sample clusters. **e**, Gene set expression score (top beeswarmplots) and enrichment among marker genes (bottom heat maps) for signatures of fibroblasts(FB), hepatic stellate cells (HSC), and vascular smooth muscle cells (VSMC), obtained from [28]. **f**, UMAP plot of mesenchymal cells from healthy and post-PVE fresh samples, colored by condition.

g, Heatmap showing proportions of each identified cell type per condition. Numbers indicate the total number of cells identified for each cluster in each condition. Source data are provided as a Source Data file for panels a, b and e.

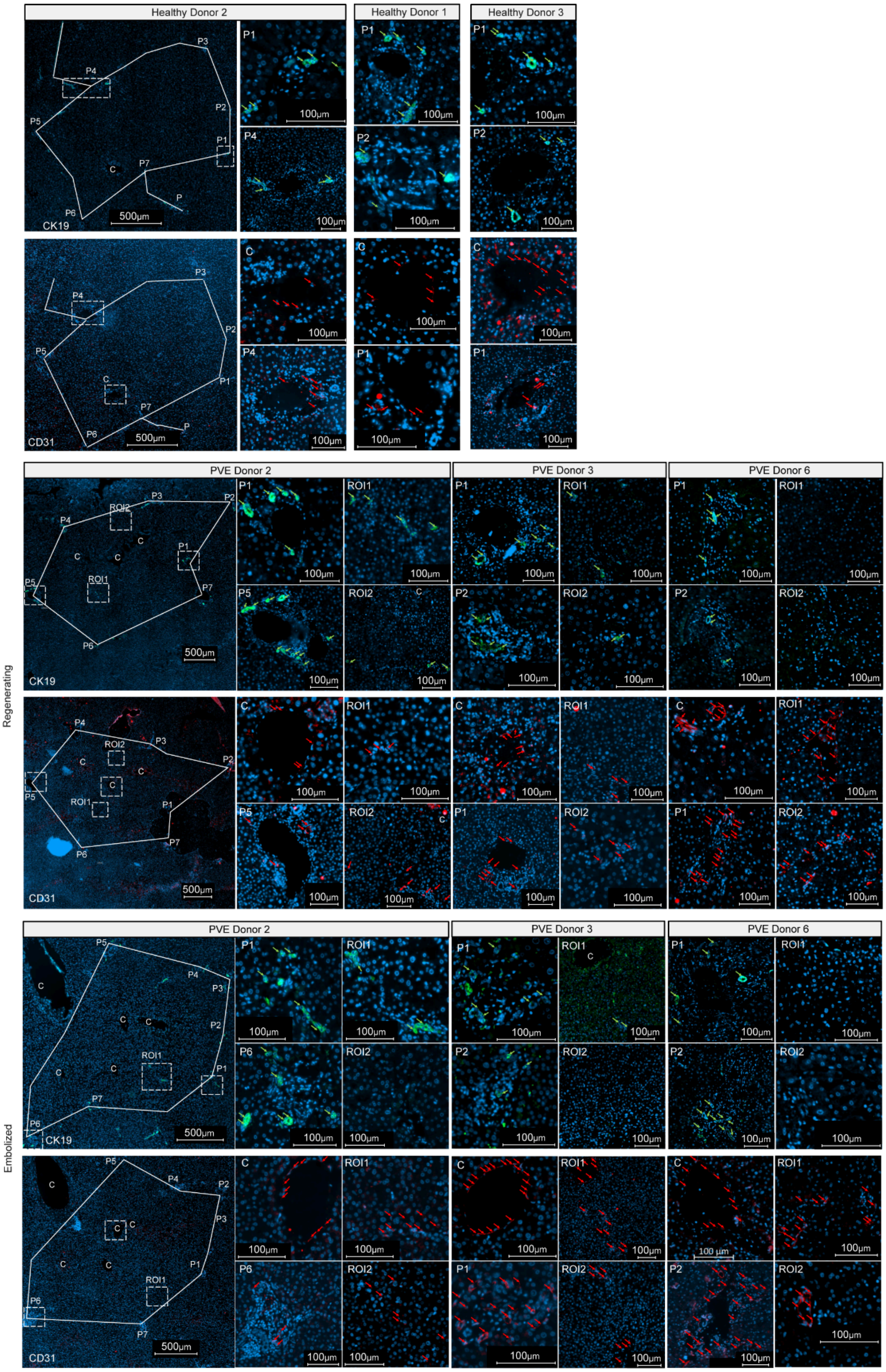

Healthy Donor 2
Healthy Donor 1
Healthy Donor 3
CK19
CD31
500µm
100µm
PVE Donor 2
PVE Donor 3
PVE Donor 6
Regenerating
Embolized

Supplementary Fig. 7 | Identification of newly formed bile ducts and vessel architecture in regenerating liver using immunofluorescence. Representative neighboring sections with stainings of CK19 (bile duct marker) and CD31 (*PECAM1*, endothelial marker) in healthy and post-PVE (regenerating and embolized) human liver tissues. Representative liver lobules highlighting central veins (C), portal fields (P) and a rough estimation for the lobular border as well as regions of interest (ROI) within the lobular parenchyma were shown for Healthy Donor 2 and PVE Donor 2. Explicit lobular features as well as ROI were shown in higher magnification demonstrating i) a higher number of bile ducts in portal fields of regenerated tissue samples, ii) CD31 positive sites co-localized with CK19 positive sites within the lobular parenchyma in regenerated tissue samples and iii) CD31 positive cells and vessel structures within the lobular parenchyma of embolized tissue samples. Arrows in green (CK19) and red (CD31) indicate positive bile and vascular cells and structures, respectively.

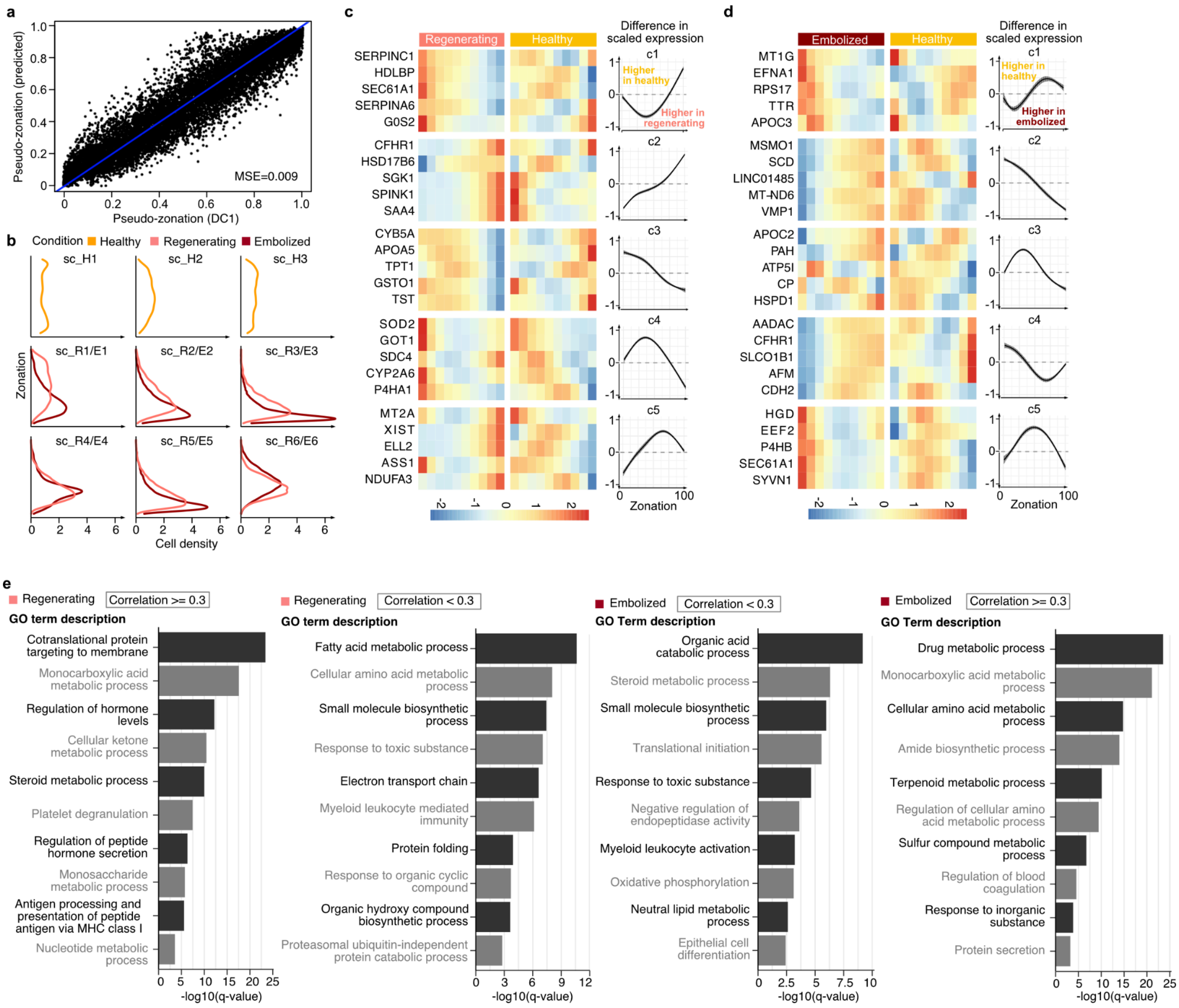

Supplementary Fig. 8 | Additional information on hepatocyte zonation inference. **a,** Scatterplot comparing original (x-axis) and predicted (y-axis) hepatocyte pseudozonation. Prediction was done based on 10-fold cross validation. **b,** Cell distribution along zonation in every condition, split by donor. **c, d**, Heatmaps showing example g enes with different zonation patterns between the healthy and regenerating (c) or e mbolized (d) tissue hepatocytes. **e,** Enriched GO Terms for the genes with fitt ed expression along pseudo zonation correlated (PCC>=0.3, top) and not correlated (PCC<0.3, bottom) between healthy and regenerating or embolized tissue hepatocytes. Source data are provided as a Source Data file for panels c, d and e.

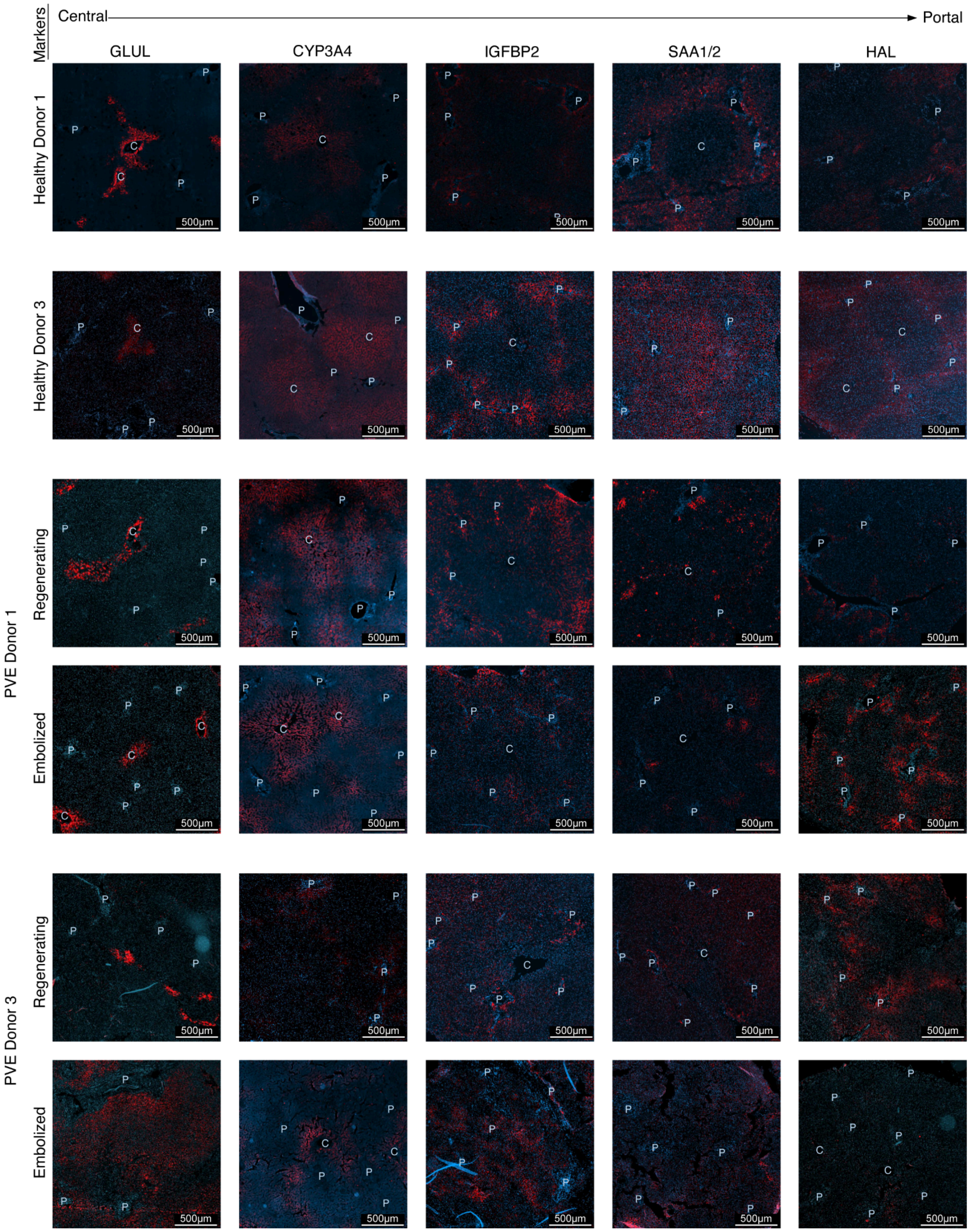

Supplementary Fig. 9 | Identification of zonation-specific marker expression in healthy and post-PVE liver tissue sections using immunofluorescence. Representative sections with stainings of central (GLUL, CYP3A4), middle (IGFBP2) and portal (SAA1/2, HAL) zonation markers in healthy and post-PVE (regenerating and embolized) human liver tissues. Additional donors to those shown in Fig. 3f.

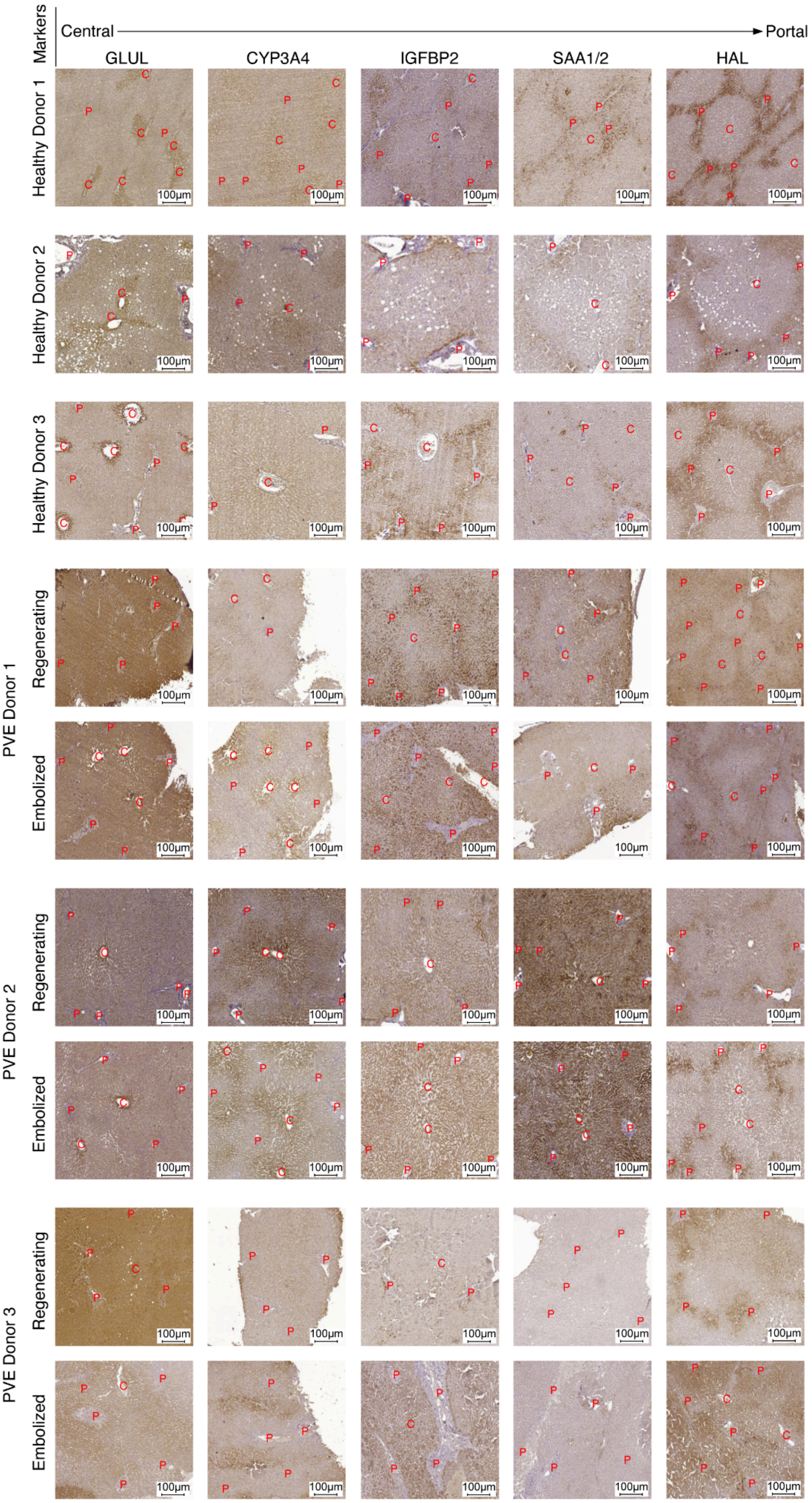
Markers
Central
Portal
GLUL
CYP3A4
IGFBP2
SAA1/2
HAL
Healthy Donor 1
Healthy Donor 2
Healthy Donor 3
PVE Donor 1
Regenerating
Embolized
PVE Donor 2
Regenerating
Embolized
PVE Donor 3
Regenerating
Embolized
100µm

Supplementary Fig. 10 | Identification of zonation-specific marker expression in healthy and post-PVE liver tissue sections. Representative sections with DAB stainings of central (GLUL, CYP3A4), middle (IGFBP2) and portal (SAA1/2, HAL) zonation markers in healthy and post-PVE (regenerating and embolized) human liver tissues. Images are shown at 5 x magnification.

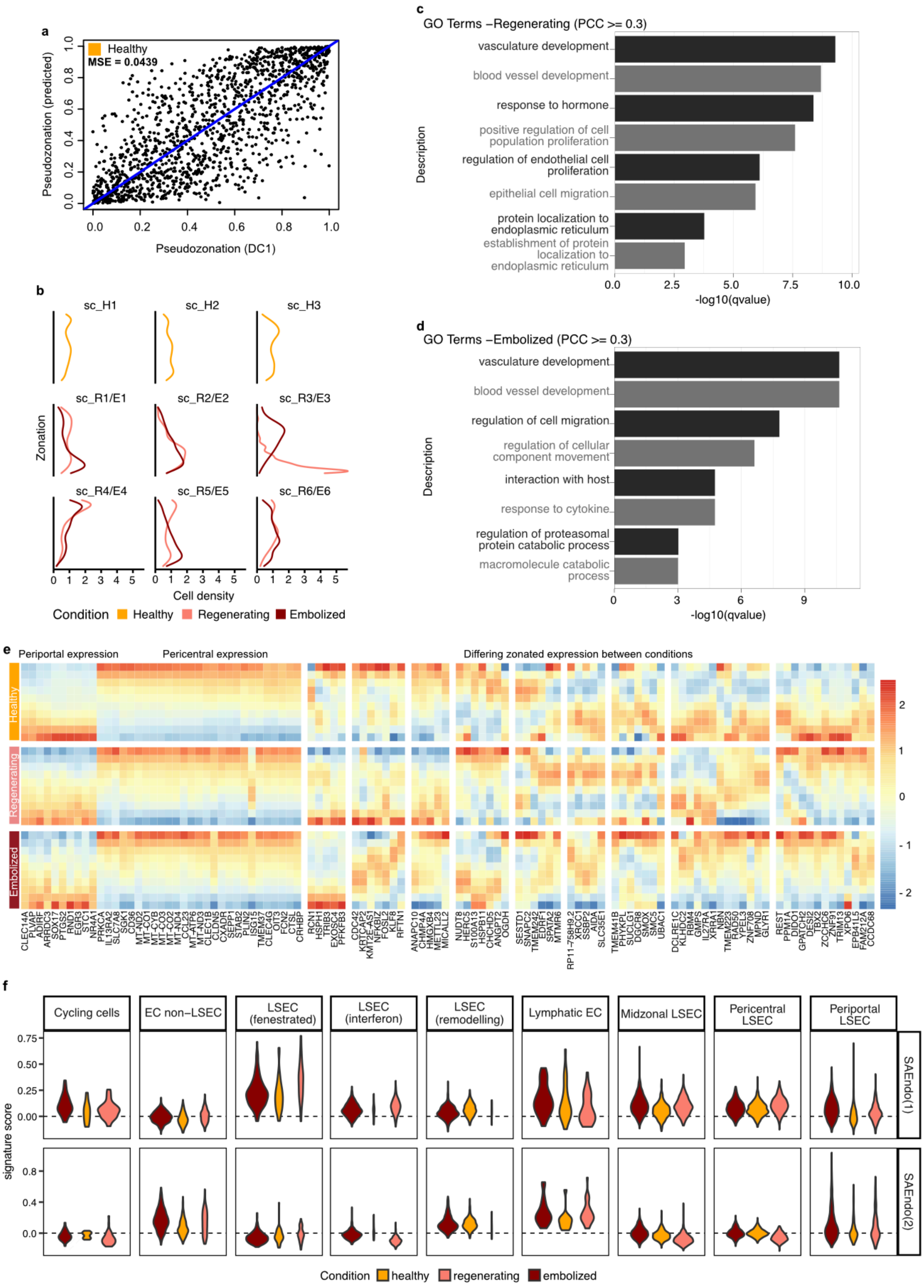

Supplementary Fig. 11 | Additional information on LSEC zonation inference.
a, Scatterplot comparing original (x-axis) and predicted (y-axis) LSEC pseudozonation. Prediction was done based on 10-fold cross validation (see Methods).
b, LSEC distribution along zonation trajectory in every condition, split by donor.
c, d, Enriched GO Terms for the

genes with fitted expression along pseudozonation correlated (PCC>=0.3, top) between healthy and regenerating (**c**) or embolized (**d**) LSECs. No terms were found enriched from non-correlated genes. **e**, Heatmap shows zonation-specific periportal, midzonal, and pericentral LSEC gene expression, grouped by similarity between conditions. Genes were chosen from the top correlated and anti-correlated genes between healthy and regenerating, and healthy and embolized conditions. **f**, Scar-associated endothelial (SAEndo) signature scores in endothelial cell populations, in each condition. Source data are provided as a Source Data file for panels b-f.

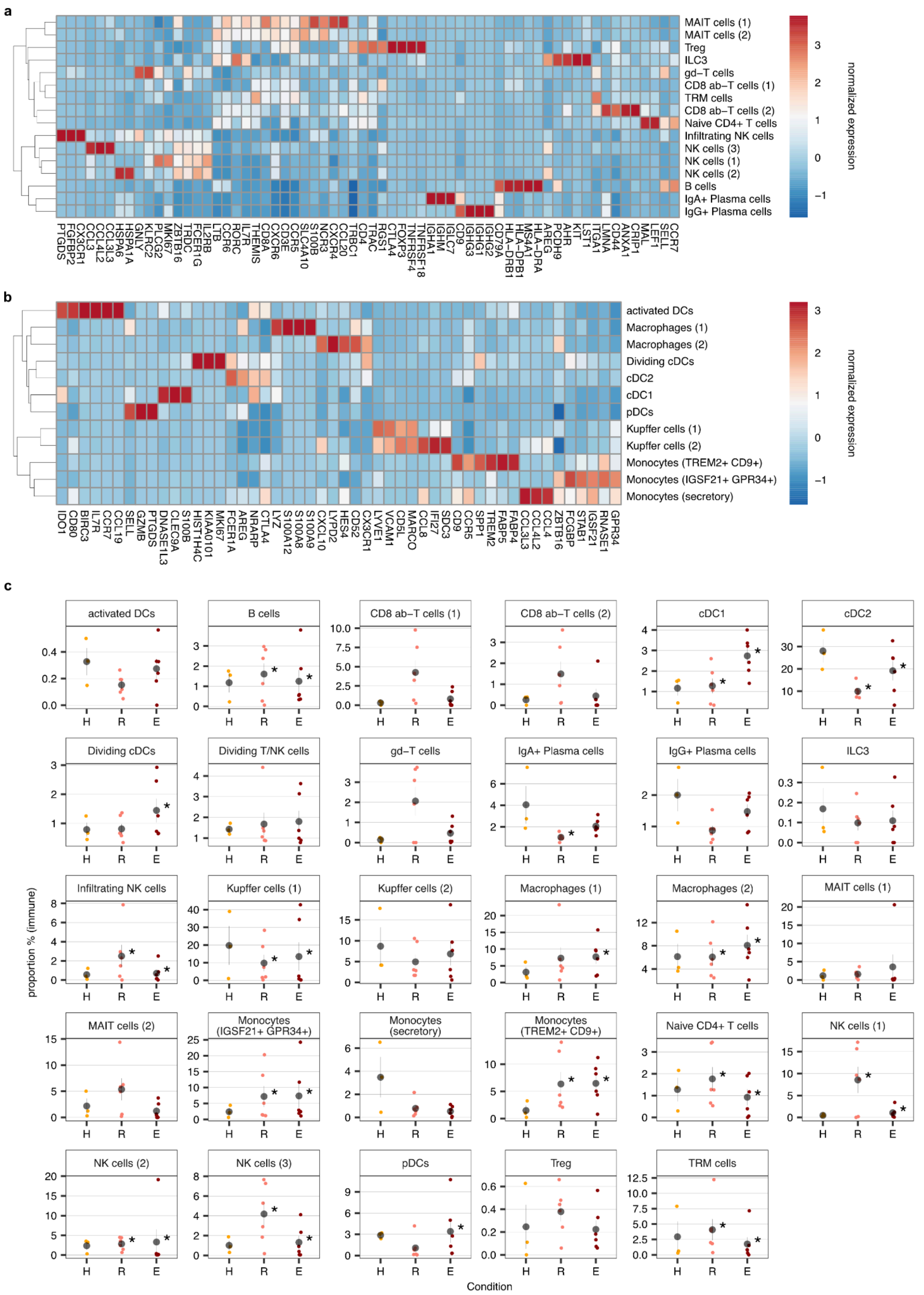

Supplementary Fig. 12 | Immune cell subtypes markers and relative cellular abundance. a, Heatmap with average gene expression for selected markers of lymphoid subpopulations. **b**, Heatmap with average gene expression for selected markers of myeloid

subpopulations. **c**, Proportion of immune cell populations per condition from the total immune cells per donor. * denotes a significant difference compared to healthy (two-tailed binomial test, $p<0.05$). H - healthy; R - regenerating; E - embolized. Source data are provided as a Source Data file for panels a and b.

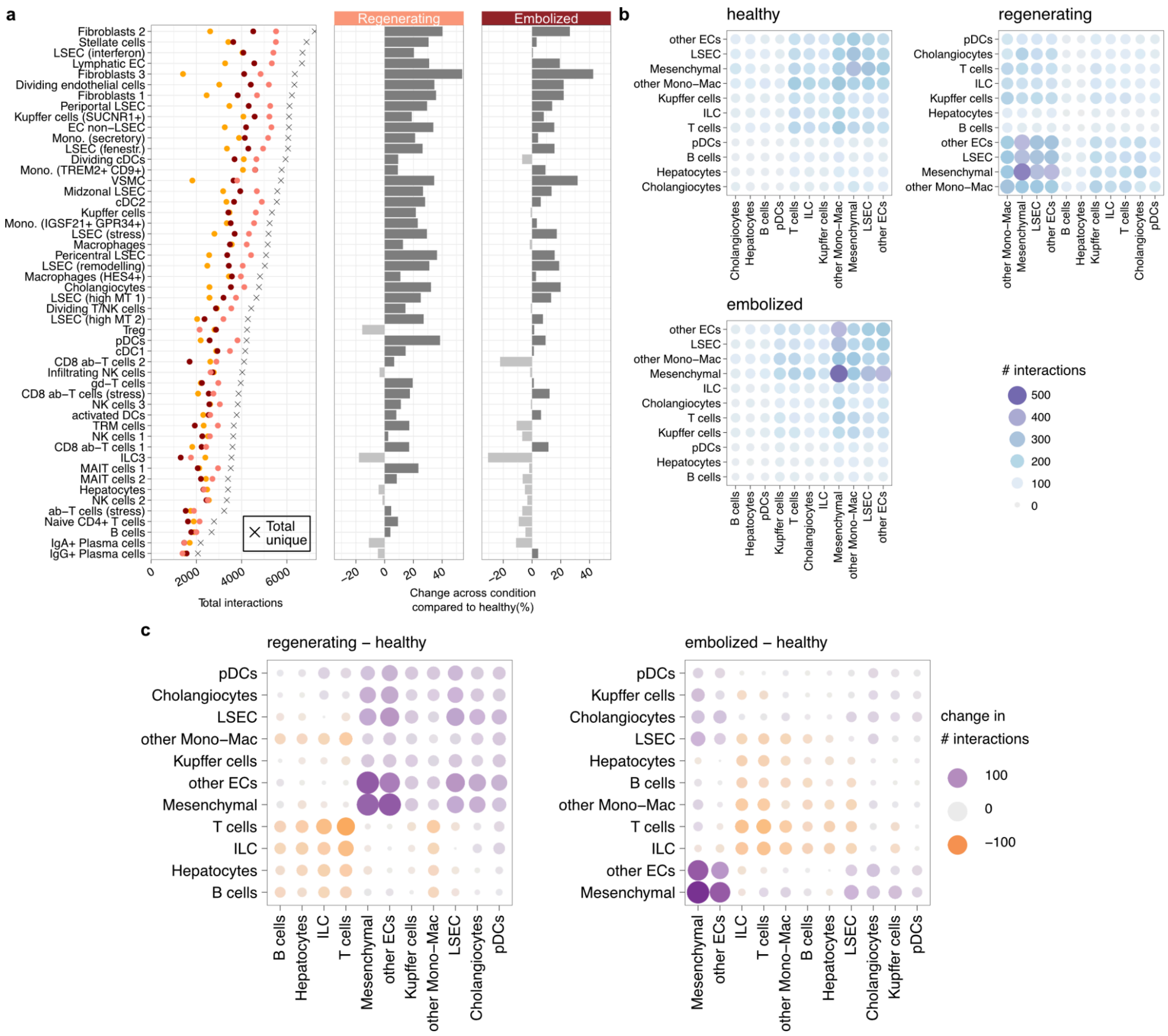

Supplementary Fig. 13 | Differences in the number of cell-cell interactions between conditions. a, Number of interactions involving each cell subtype, in each condition and in total (left dotplot), along with variation in the number of interactions between healthy and regenerating (middle) or embolized (right) condition. **b**, Absolute number of interactions per condition involving each major cell type pair. **c**, Difference in number of interactions for each pair of major cell types between healthy and regenerating (left) or embolized (right) condition. Source data are provided as a Source Data file for panels a-c.

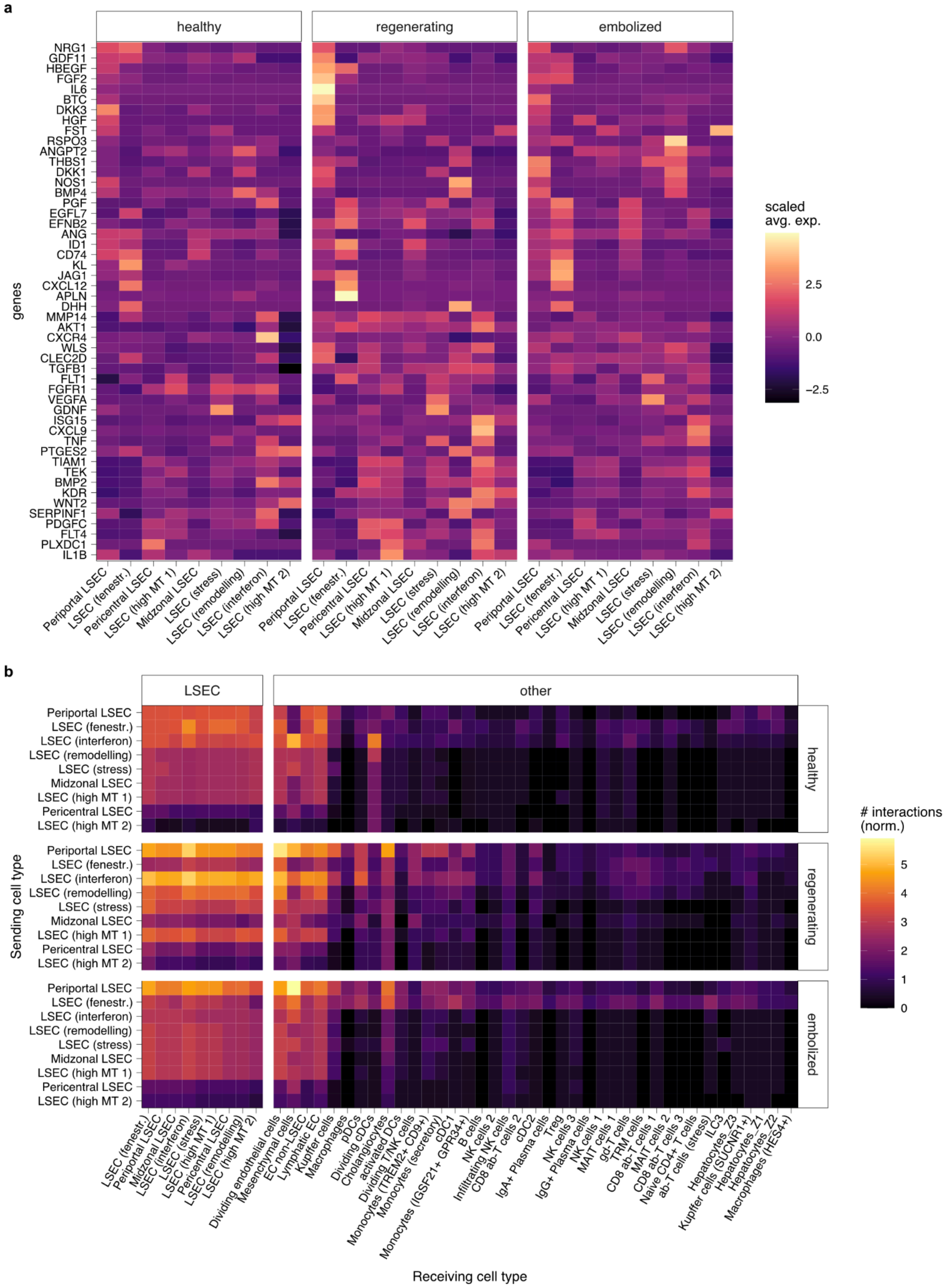

Supplementary Fig. 14 | Cell-cell interactions involving previously reported genes related to angiocrine function. a, Expression of reported angiocrine genes (compiled from [67-70]) in LSEC cell subtypes. **b**, Normalized number of cell-cell interactions involving at least one angiocrine gene.

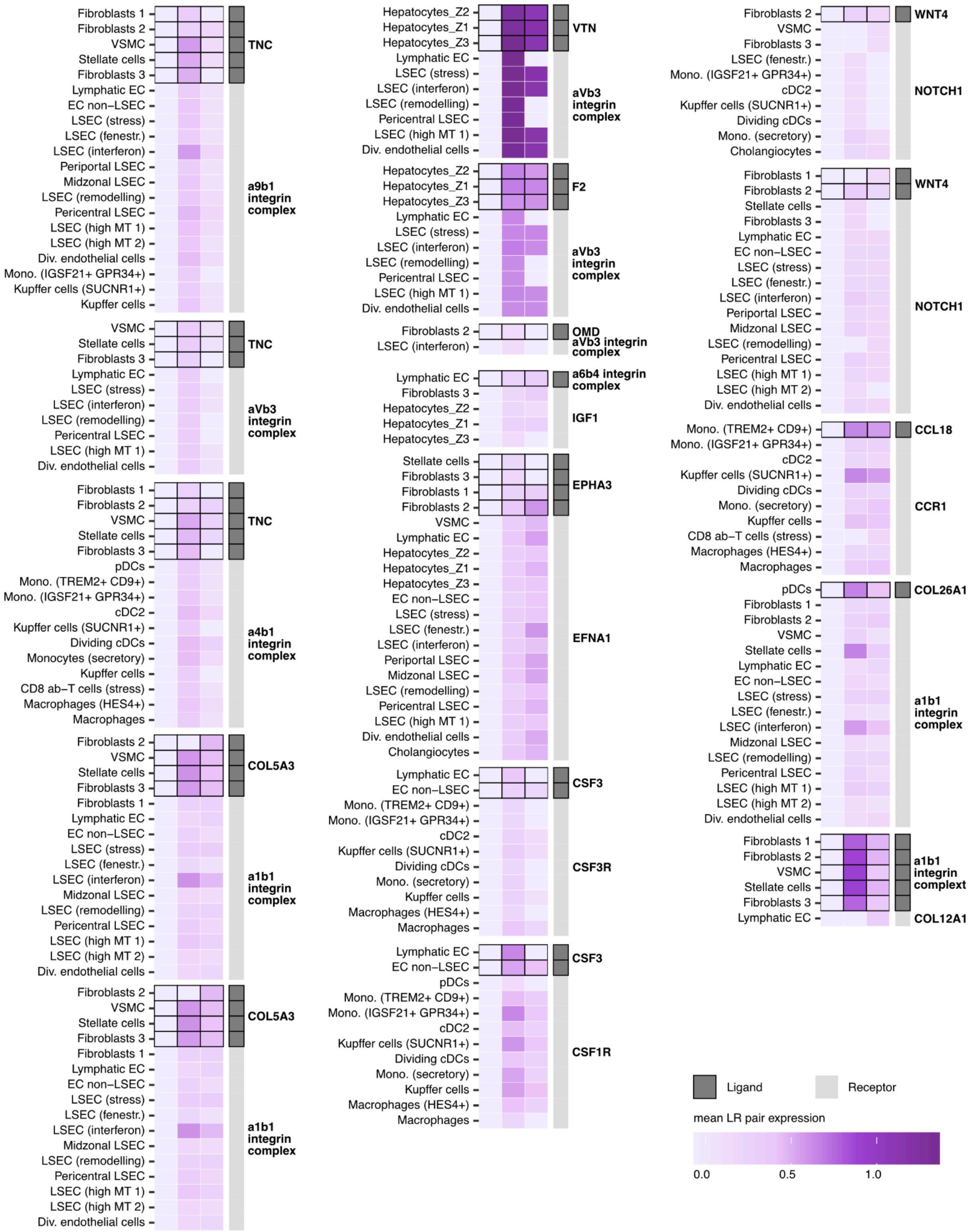

Supplementary Fig. 15 | Additional heatmaps showing mean expression for selected ligand-receptor pairs present in regenerating or embolized tissue. Black outline denotes the cell type uniquely expressing the ligand. Gray sidebar distinguishes expression of ligand and receptor.

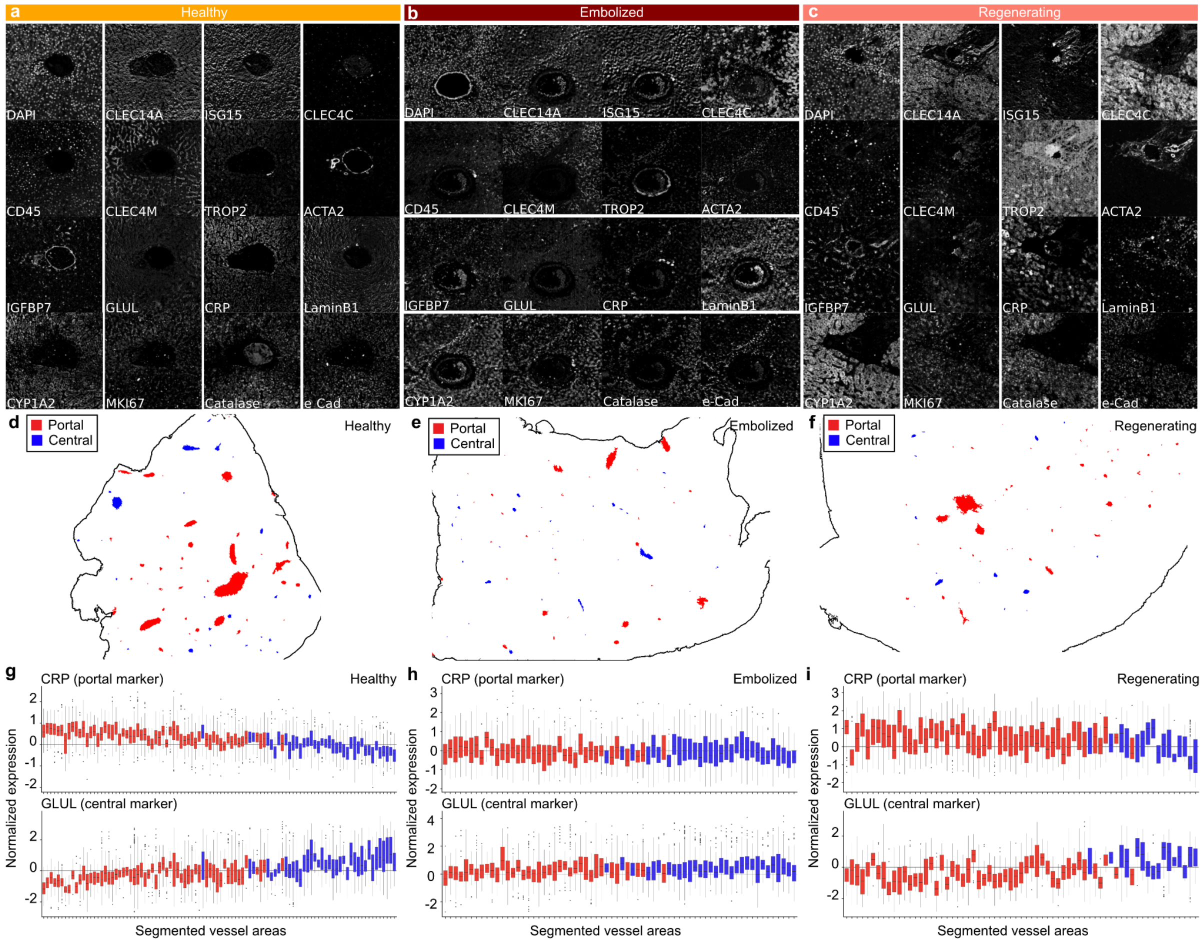

Supplementary Fig. 16 | Multiplexed immunohistochemistry and vessel area segmentation. a-c, Tissue section showing individual stainings obtained from the 4i protocol, for each condition. **d-f**, Inferred portal and central vessel areas for healthy, embolized, and regenerating samples. **g-i**, Boxplots showing normalized expression of hepatocyte periportal (top) and pericentral (bottom) markers in hepatocytes from the microenvironment of each detected vessel. Boxplots are colored based on the type of vessel inferred. Boxplot shows the median (center line), 25th, and 75th percentile (lower and upper boundary), and the whiskers indicate the 1.5x inter-quartile range, with outliers shown as individual data points. Source data are provided as a Source Data file for panels g-i.

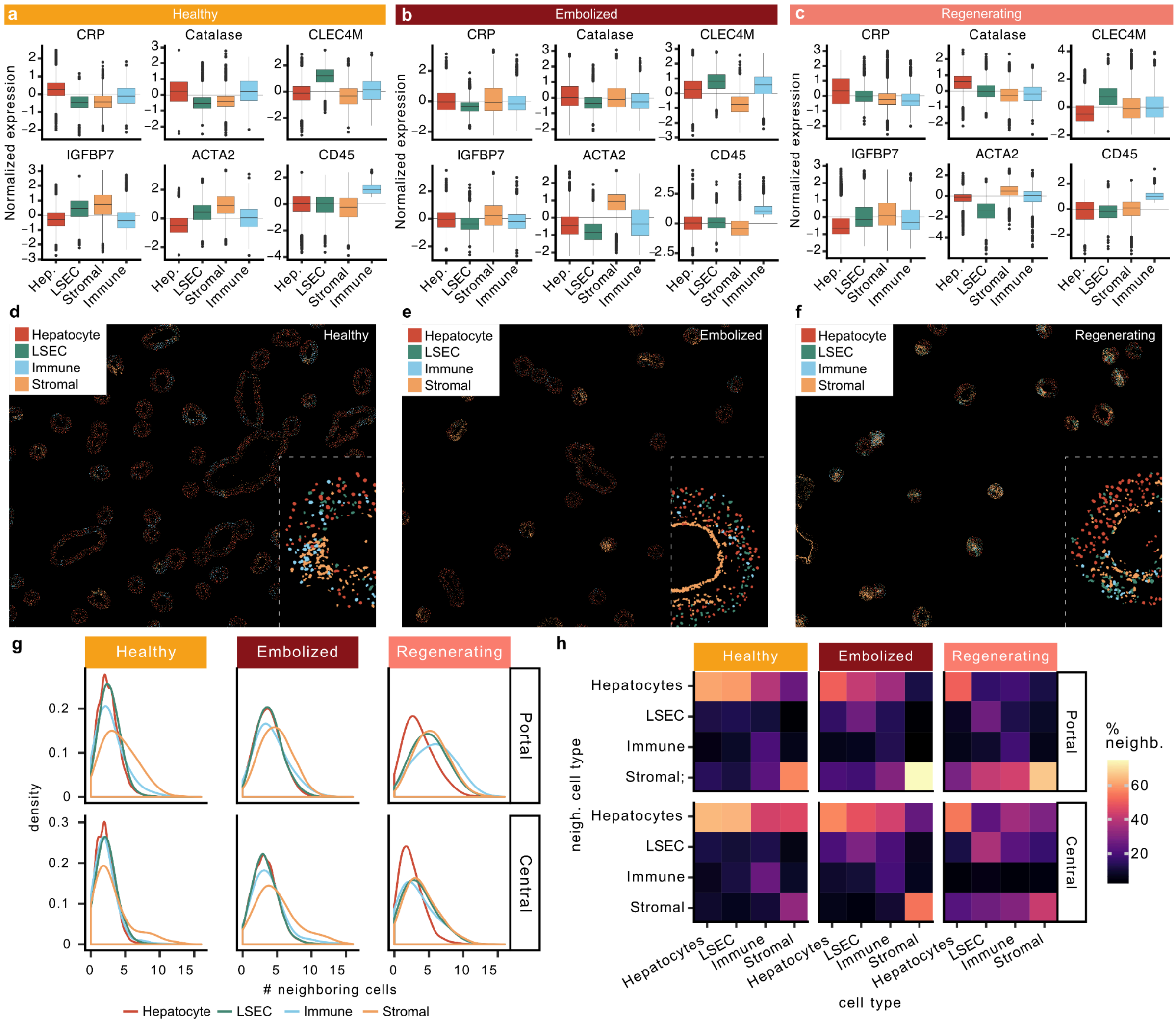

Supplementary Fig. 17 | Vessel area cell type analysis from multiplexed IHC. a-c, Normalized fluorescence of selected markers used to identify the major cell types in the 4i data. **d-f**, Large area view of vessel microenvironments with segmented nuclei colored by the annotated major cell type. **g**, Density plots showing the total number of neighboring cells per cell type (color), vessel type (rows) and condition (columns). **h**, Proportion of neighboring cell types (columns) that each cell type (rows) has, per vessel type and condition. Source data are provided as a Source Data file for panels a-c, and g, h.
