# Supplementary material for: Cell atlas of the regenerating human liver after portal vein embolization
Source: Nat Commun. 2024 Jul 11;15:5827. doi: 10.1038/s41467-024-49236-7 (PMC11239663; doi:10.1038/s41467-024-49236-7)
Supplement: Supplementary file 3 — Description of additional supplementary files [file 41467_2024_49236_MOESM3_ESM.pdf]

Description of Additional Supplementary Files

Supplementary Data 1 - Information about liver donors and experiments. Donors of healthy tissues include information of associated medical conditions. Donors of post-PVE samples include information on time span of PVE.

Supplementary Data 2 - Number of detected cell types across donors in sn RNA-seq datasets.

Supplementary Data 3 - Number of detected cell types across donors in sc RNA-seq datasets.

Supplementary Data 4 - Differentially expressed genes between healthy and embolized or regenerating conditions in each of the major cell types studied.

Supplementary Data 5 - Differential zonation between healthy and regenerating or embolized tissue hepatocytes.

Supplementary Data 6 - Differential zonation between healthy and regenerating or embolized tissue liver sinusoidal endothelial cells.

Supplementary Data 7 - Enriched types of variable interactions per medical condition.

Supplementary Data 8 - Information about sc RNA-seq experiments and summary metrics.

Supplementary Data 9 - Unconjugated primary as well as secondary antibodies for immunohistochemical stainings of liver lobule zonation markers. Summary of antibodies including antigen, conjugate, isotype and host species, dilution, as well as incubation time and temperature.

Supplementary Data 10 - Sample information on the 10x Genomics used chemistry and Cell Ranger version.

Supplementary Data 11 - Thresholds for quality-control filtering the scRNA-seq samples.

Supplementary Data 12 - Information about sn RNA-seq experiments and summary metrics.
